# Risk of gestational diabetes mellitus in systemic lupus erythematosus pregnancy: a systematic review and meta-analysis

**DOI:** 10.1186/s12884-019-2329-0

**Published:** 2019-05-22

**Authors:** Yuanyuan Dong, Ziwei Dai, Zhihui Wang, Hong Wang, Feifei Yuan, Ying Zhu, Dongqing Ye, Bin Wang

**Affiliations:** 1 Department of Epidemiology and Biostatistics, School of Public Health, Anhui Medical University, 81 Meishan Road, Hefei, 230032 Anhui China; 2 The Key Laboratory of Major Autoimmune Diseases, Anhui Medical University, 81 Meishan Road, Hefei, 230032 Anhui China; 3 Department of Obstetrics and Gynecology, the First Affiliated Hospital of Anhui Medical University, Hefei, 230032 Anhui China

**Keywords:** Systemic lupus erythematosus, Pregnancy, Glucocorticoids, Gestational diabetes mellitus

## Abstract

**Background:**

It is well established that the risks of insulin resistance and diabetes mellitus are elevated in systemic lupus erythematosus (SLE) patients. However, the relationship between SLE pregnancy and gestational diabetes mellitus (GDM) is still obscure. We perform the present systematic review and meta-analysis to determine the relationship between GDM and SLE pregnancy.

**Methods:**

According to the Preferred Reporting Items for Systematic Reviews and Meta-Analyses (PRISMA) statement, relevant studies were carefully retrieved through PubMed, Cochrane library and Web of Science, China National Knowledge Infrastructure, Wanfang database and China Biology Medicine database from inception till 30 August 2018. GDM risk ratio (RR) of pregnant SLE patients versus controls was calculated to evaluate the association between GDM and SLE. Pooled RRs and 95% confidence intervals (CIs) were calculated using random effects model by R software.

**Results:**

The literature retrieval identified 339 potential studies in total, and five studies containing 3432 pregnant participants with 248 GDM events were included finally. Pooled analysis found that the risk of GDM were not significant increased in SLE patients compared to controls (RR = 1.08, 95% CI = 0.49 to 2.41, Z = 0.19 and *P* = 0.848). Nevertheless, meta-regression identified that glucocorticoids use and anti-double stranded DNA antibodies positive of SLE patients were positively associated with the risk of GDM.

**Conclusions:**

Our meta-analysis demonstrated that SLE pregnancy may not increase the risk of GDM, but the steroid use during pregnancy was associated with increased risk of GDM. Further large prospective and basic immunologic studies should be implemented for exploring the mechanism underlying glucocorticoids use and GDM.

**Electronic supplementary material:**

The online version of this article (10.1186/s12884-019-2329-0) contains supplementary material, which is available to authorized users.

## Background

Systemic lupus erythematosus (SLE) is a chronic autoimmune disease predominantly affecting women of reproductive ages, characterized by loss of self-tolerance, production of autoantibodies and deposition of antigen-antibody complexes. SLE usually involved multiple organs, like skin, joints, kidneys and nervous system. SLE is reported to involve multiple organs like skin, joints, kidneys and nervous system. Besides, pregnancy is also dangerous for women with SLE. Recently, more studies focus on the higher risks of pregnancy complications in SLE patients, especially in patients with the existence of autoantibody positive, antiphospholipid syndrome (APS), nephritis or pulmonary hypertension, and patients with immunosuppressive therapies [1]. Although the fertility rate of SLE women is normal, SLE pregnancy was reported to significant increased risk of adverse maternal and fetal adverse outcome [2]. Increasing numbers of studies reported that autoimmune diseases including SLE could affect the process of pregnancy and induce adverse pregnancy outcomes (APOs), such as gestational hypertension, pre-eclampsia and lupus flares [3–6]. Gestational diabetes mellitus (GDM) is the most common autoimmune endocrine complication of pregnancy. However, the relationship between GDM and SLE has not been well illustrated yet.

GDM is a common pregnancy complication, defined as impaired glucose tolerance first detected during pregnancy, and is associated with adverse maternal and fetal outcomes, including hyperinsulinemia, hypocalcemia, hyperbilirubinemia, preeclampsia, and macrosomia [7, 8]. The risk of dystocia, usually caused by macrosomia, cesarean section and even stillbirth are also increased in pregnancy women with GDM [9, 10]. For the long-dated consequences, GDM is related to markedly increased risk of post-partum diabetes and cardiovascular diseases [7, 11]. Although the exact pathological mechanism underlying GDM is still obscure, exacerbated insulin resistance is reported to play a pivotal role in GDM. SLE is also reported to associate with increased risk of insulin resistance and diabetes mellitus. Besides, studies also indicated that abnormal insulin secretion may be caused by autoimmune damage [12], and autoimmune response may impair the function of pancreatic beta cell [13]. Patients with SLE are accompanied with autoimmune microenvironment during pregnancy, which may relate to GDM incidence. Present studies indicated that SLE pregnancy is associated with elevated risk of GDM [10]. However, studies also reported that the GDM risk of SLE patients was not significant increased [9, 14, 15]. Besides, whether there are specific risk factors influencing GDM incidence in SLE patients is also doubtful. In light of these facts, we performed this study to comprehensively review and meta-analyze the relationship between SLE and GDM.

## Methods

This study was performed was conducted according to the Preferred Reporting Items for Systematic Reviews and Meta-Analyses (PRISMA) statement [16] (see Additional file 1: Table S1) .

### Review question

According to the PICOs scheme, the review question of our meta-analysis was whether pregnant women with SLE (Participants) were associated with increased risk ratio (RR) of GDM (Outcomes) compared with non-SLE pregnant women (Comparisons) of cohort and case-control studies (Study designs).

### Search strategy and study selection

PubMed, Web of Science, and Cochrane Library were comprehensive searched for relevant studies from inception to 30 August, 2018. MeSH terms and Web of Knowledge topics were used. The detailed search strategy was provided in Additional file 1: Figure S1 To identify the potential grey literatures, we also searched relevant studies of our analysis in three Chinese databases, China National Knowledge Infrastructure, Wanfang database and China Biology Medicine database. Furthermore, bibliographies from eligible original studies and reviews were also searched manually.

The two reviewers (Yuanyuan Dong, Ziwei Dai) firstly performed an initially title and abstract screening independently. Studies fully agreed on eligible for inclusion by two reviewers will be further assessed for availability through full-test review. Eligible studies must respectively fulfill the inclusion criteria as following: a) article reported the incidence risk of GDM in SLE pregnant women compared with controls; b) article reported original data eligible for calculate of the index; c) SLE was diagnosed according to ACR criteria. d) GDM was defined as any degree of glucose intolerance with onset or first detected during pregnancy or diagnosed as included studies reported criteria [17]; e) original research designed as case-control or cohort study. When necessary, we contacted corresponding authors for full-text or relevant data. Additionally, the studies in the forms of case reports, clinical trials and reviews were excluded. There was no language restriction. To avoid including data of duplicate publication, only most comprehensive study including more abundant data was analyzed.

### Methodological quality assessment and data extraction

Data extraction and methodological assessment were conducted by two investigators (Yuanyuan Dong, Ziwei Dai) independently and confirmed by a third reviewer (Zhihui Wang). The Newcastle-Ottawa Scale (NOS) for cohort and case-control studies were used to assess the quality [18]. For further exploring the impact of SLE on GDM risk, the following characteristics were also extracted from included studies: publication year, first author’s name, region, disease duration, diagnosis criteria of GDM and SLE, study design, sample size, age of pregnant women of SLE patients and controls. In addition, some clinical characteristics of SLE, like proportions of antiphospholipid antibody (aPL) positive, anti-double stranded DNA antibodies (anti-ds-DNA) positive, lupus nephritis, and hydroxychloroquine or glucocorticoids use () were also obtained. Any discrepancy in processes of literature search, study selection, quality assessment or original data extraction was resolved with consensus via discussing.

### Statistical analysis

Original data regarding GDM risk of SLE patients compared to controls was used to calculate the effect size. The relationship between SLE and GDM was evaluated by RR and 95% confident interval (CI) according to either fix effect model or randomized effects model, depending on the heterogeneity among included studies. Cochran’s Q statistic was used to assess between studies heterogeneity; besides, *I*^*2*^ test was also used to quantify the degree of inconsistency by calculating the percentage of total variation between studies, where due to heterogeneity rather than chance. Heterogeneity graded by *I*^2^ was set as low (< 25%), medium (25–75%) and high (≥ 75%) [19]. If there was a significant heterogeneity, the random effects model is chosen, otherwise the fix effect model is chosen [20, 21], visualized by forest plots. Sensitivity analysis was performed to validate the stability of the meta-analysis by consecutively excluding each enrolled study. Any *P* < 0.05 was considered as statistically significant, and all statistical analyses were conducted by R software (R Foundation for Statistical Computing, Vienna, Austria).

## Results

### Search results

**Fig. 1:**
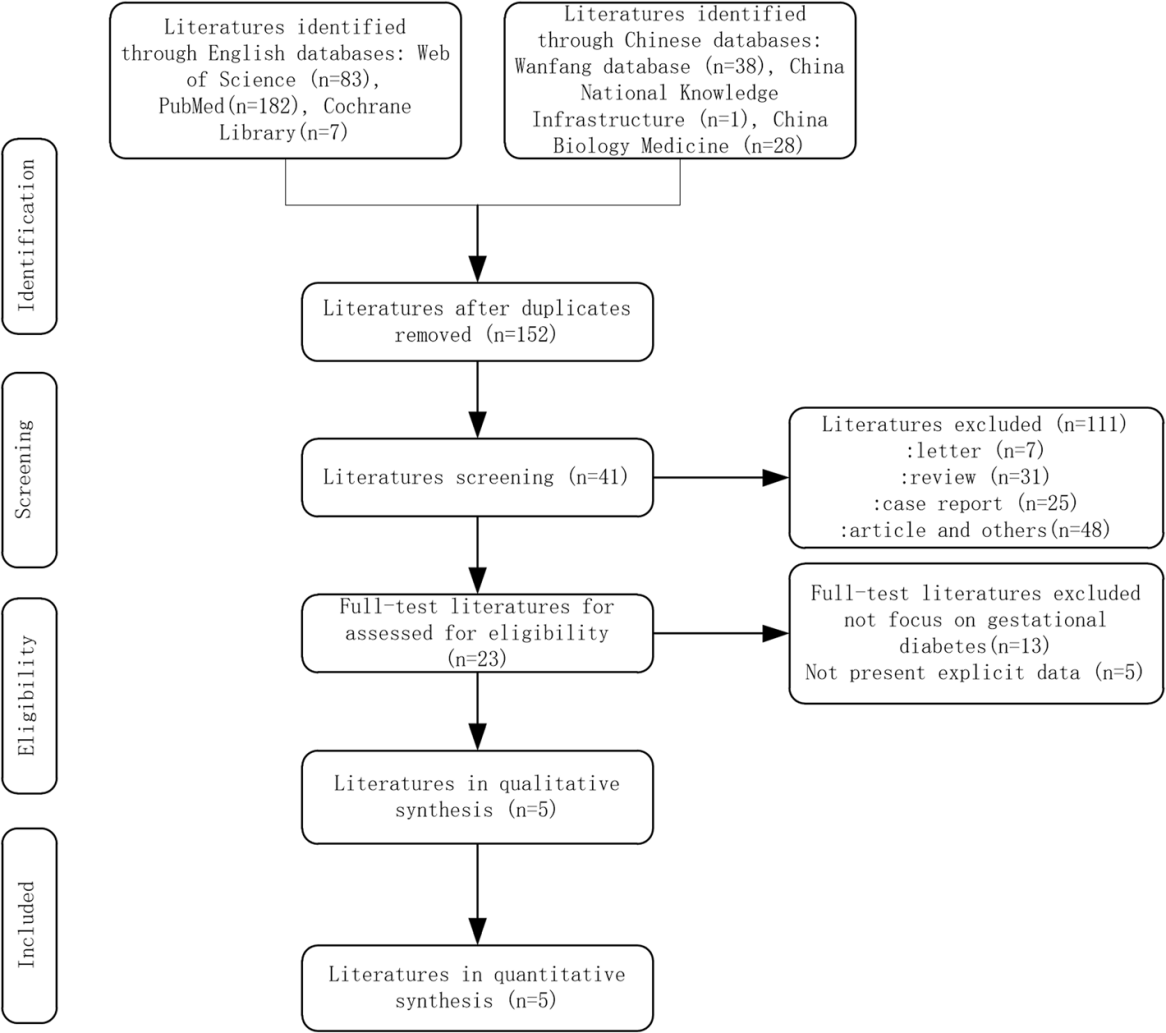
Flow chart of the literature search and study selection

This search identified 339 references totally from PubMed (*n* = 182), Web of Science (*n* = 83), Cochrane Library (*n* = 7), China National Knowledge Infrastructure (n = 1), Wanfang database (*n* = 38) and China Biology Medicine database (*n* = 28) (Fig.1). After removal of duplicates and screening of title and abstract, a total of 23 records were obtained for full-text review. No any additional study was retrieved from references search. At last, five studies containing 714 SLE patients and 2718 controls were eligible for inclusion. In addition, the NOS scores of all included studies were acceptable detailed in Table 1. Nevertheless, methodological flaws were still existent. First, the sample sizes of some studies were small [15, 22]. The diagnosis criteria for APOs including GDM were obscure [14, 15, 22]. Besides, factors as previous pregnancy history of SLE patients and controls were not well controlled.

**Table 1:** Characteristics of included studies

Author	Country	Year	N of cases	N of controls	Average age of cases^a^	Average age of controls^a^	Disease duration^a^	Study type	Diagnosis criteria for SLE	Results of quality assessment
Wu J [9]	China(A)	2018	338	1014	29.5	29.7	5.6	Retro-cohort	ACR	8
Abdwani R [10]	Oman(A)	2018	56	91	31.0	29.0	NA	Retro-cohort	ACR	8
Phansenee S [14]	Thailand (A)	2017	133	1394	29.5	27.4	NA	Retro-cohort	NA	7
Galappatthy P [15]	Sri Lanka (A)	2017	79	85	25.9	NA	8.0	Retro-case-control	ACR	7
Yan Yuen [22]	Canada	2008	108	134	42.0	38.0	15.0	Retro-case-control	ACR	8

^a^The unit is year; *A* Asia, *ACR* American College of Rheumatology criteria, *N* Number, *Retro* Retrospective, *NA* Not available, *Y* Year

### Characteristics of studies

The baseline characteristics of five eligible studies [9, 10, 14, 15, 22] were listed in Table 1 and Additional file 1: Table S2 Except for study by Yan Yuen [22], included studies were all conducted in Asia. Four studies reported that SLE was diagnosed according to ACR criteria, and study by Phansenee S et al. has not reported SLE diagnostic criteria [14]. Two studies have not indicated the disease duration of SLE patients [10, 14]. All the included studies were retrospectively designed. Two studies were case-control designed [15, 22] and three studies were cohort designed [9, 10, 14].

Wu J et al. reported significantly decreased risk of GDM in SLE patients than controls (5.6% vs 11.5%) [9], whereas research by Abdwani R et al. [10] reported that the GDM risk of SLE patients were increased. The remaining three articles have not found any significant difference of GDM risk between SLE patients and controls [14, 15, 22]. Four studies reported the proportions of patients used hydroxychloroquine and glucocorticoids during pregnancy [9, 10, 14, 15]. Notably, in research of Wu J et al. [9], about 97.6% of SLE patients used glucocorticoids during pregnancy.

### RR of GDM in SLE pregnancy

**Fig. 2:**
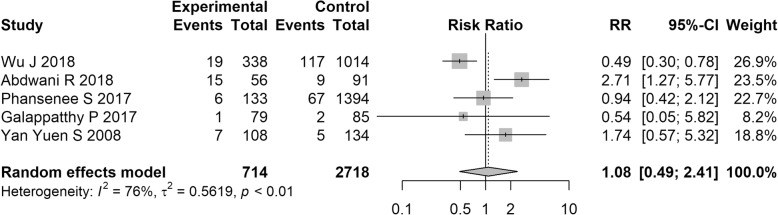
Forest plots of the RR of GDM in SLE pregnancy

In total, five studies containing 714 SLE pregnant women reported the RR of GDM between 5 and 28.3%. Meta-analysis combining the five studies yield that the pooled RR of GDM was 1.08 (95% CI = 0.49 to 2.41, Z = 0.19 and *P* = 0.848) in SLE patients with significant heterogeneity (*I*^*2*^ = 76%; Tau^2^ = 0.56, *P* < 0.01) (see Fig. 2). Meta-regression on factors of sample size, age of cases, publication year, and proportions of aPL positive, nephritis and hydroxychloroquine use indicating that none of these factors was the source of heterogeneity. However, glucocorticoids use (r = 0.0024, *P* = 0.002) and anti-ds-DNA positive (r = 0.0022, *P* = 0.003) of SLE patients were reported to positively associate with GDM risk (see Table 2). Sensitivity analysis indicated that the result of meta-analysis was stable whatever which study was omitted (Additional file 1: Figure S2). Because we have five included studies, the evaluation of publication bias was not necessary.

**Table 2:** Meta-regression analysis coefficients of risk ratio of GDM

Variables	Coefficient (SE)	95% CI	*Z*	*P*
Publication year	−0.0028 (0.0108)	[− 0.0238, 0.0183]	−0.2557	0.800
N of cases	0.0004 (0.0002)	[<−0.0000, 0.0008]	1.8972	0.058
Age of cases	0.0034 (0.0078)	[−0.0120, 0.0187]	0.4334	0.665
aPL	−0.0066 (0.0063)	[−0.0190, 0.0057]	−1.0529	0.292
anti-ds-DNA	0.0022(0.0007)	[0.0008,0.0037]	3.0053	0.003*
Lupus nephritis	−0.0023 (0.0044)	[−0.0109, 0.0064]	− 0.5146	0.607
HCQ use	0.0021 (0.0011)	[<−0.0000, 0.0043]	1.9213	0.055
Glucocorticoids use	0.0024 (0.0008)	[0.0009, 0.0040]	3.0512	0.002*

aPL, antiphospholipid antibodies; anti-ds-DNA, anti-double stranded DNA antibodies; HCQ, hydroxychloroquine; CI, Confident interval; N, Number; SE, Standard error; *, *P* < 0.05

## Discussion

Women with SLE are receiving better multidisciplinary antenatal care ensuring better pregnancy outcome with the development of medicine. Nevertheless, current studies [5, 23–25] also reported that SLE was associated with APOs including pre-eclampsia, pregnancy-induced hypertension, spontaneous abortion. The present study have synthesized current published studies regarding GDM and SLE, and the results indicated that SLE with not associated with GDM. The potential reasons for previous inconsistent results are listed as following.

With abnormal insulin resistance playing an irreplaceable role, GDM was reported to associate with clinical factors in SLE patients. A previous meta-analysis [24] regarding the risk of maternal and fetal outcomes in SLE pregnancy identified that several factors included lupus nephritis [26, 27], aPL positive and APS [28] were responsible for the higher risk of APOs following pregnancy. We also conducted meta-regression analysis on corresponding factors to assess their impact on GDM risk. As previous studies reported that diabetes mellitus was developed around 12% of SLE patients due to high-dose glucocorticoid therapy (prednisolone use ≥1 mg/kg/day) [29, 30]. Our study also identified that glucocorticoid use during pregnancy was positively associated GDM risk in SLE patients. Consistent with our results, studies also reported that women with higher dose of steroid during pregnancy have an increased risk of diabetes, and steroid exposure should be restricted to a minimum during pregnancy [4, 31]. Glucocorticoid was known to necessary for the control of SLE disease activity during pregnancy. One original study reported that the higher risk of GDM in SLE patients compared with controls may due to high rate of glucocorticoids use of SLE patients during pregnancy [10]. Our results also demonstrated that hydroxychloroquine use and the number of cases were borderline significant factors positively associated with the GDM risk. However, hydroxychloroquine use may reduce glucocorticoid doses during pregnancy, consequently reducing the risk of GDM development. Studies also reported that hydroxychloroquine is safe enough to continue throughout the whole pregnancy process by all pregnant women with SLE [24, 32]. Simultaneously, epidemiologic studies have demonstrated that hydroxychloroquine could significantly reduce diabetes mellitus risk of SLE patients in a dose-dependent manner [33, 34]. So we hypothesized that the increased risk of GDM by hydroxychloroquine use was a false positive result. Hydroxychloroquine use during SLE pregnancy could be recommended for reducing glucocorticoid doses. The potential reason may be that medication use demands of SLE patients were differential across studies populations depending on disease activity. The hydroxychloroquine use rate was associated with glucocorticoids use rate in some extent. Besides, the glucocorticoids use rate of SLE patients during pregnancy was extremely high. The false positive result may due to the confounding of glucocorticoids. We also found that SLE patients with anti-ds-DNA were associated with higher risk of GDM. Whether it is the consequences of autoimmune dysfunction contributing to autoimmune GDM is still obscure. In words, we hypothesized that the use of glucocorticoids may increase the risk of GDM and the autoimmune dysfunction of SLE may related to autoimmune GDM.

Another reason accounting for the inconsistent results of original studies may be related to the inconsistent diagnosis criteria of GDM. As the most common metabolic disturbance among pregnant women, GDM has a series of diagnosis criteria [32, 33]. A recent meta-analysis by Behboudi-Gandevani S and colleagues reported that the worldwide prevalence of GDM was 4.4%, regardless of type of screening threshold categories. According to seven different diagnosis criteria, subgroup analysis of the study indicated that the prevalence of GDM was ranged from 2.2 to 10.6% [29]. The diagnosis criteria of GDM in original studies were different, because of the lack of international consistently diagnosis criteria for GDM. GDM risk of SLE patients is not the primary outcome of most included studies, so the screening methods of GDM have not been exactly reported. In our included studies, only two studies reported that GDM was defined as any degree of glucose intolerance with onset or first recognition during pregnancy [9, 10]. It is hard for us to evaluate the impact of criteria on GDM risk with the present available data. The inconsistent diagnosis criteria of GDM in original studies have indeed made some certain effects on the final diagnosis of GDM, which may partly account to the significant heterogeneity of our study.

Autoimmune GDM is a subset of GDM with the representation of various autoimmune antibodies (GADA, IA2-A, IAA, ZnT8-A), and account for about 10% of all GDM [13]. Autoimmune GDM was also reported to associate with higher risk of type 1 diabetes or latent autoimmune diabetes in adult. Therefore patients with autoimmune GDM was worthy of studying for the prevention of type 1 diabetes in pregnancy or afterwards [30]. SLE is an autoimmune dysfunction disease and characterizing with the presence of various antibodies, likely to associate with autoimmune GDM. Regrettably, of studies, none of the present studies, researching the relationship between SLE and GDM, reported the results of autoimmune GDM or related antibodies. Further studies focus on the relationship between autoimmune GDM and SLE are also necessary.

The major strength of this present study is that we have integrated existing research using systematic quantitative methods, minimizing the selection and reporting biases. There are also some limitations existing. First, the small sample sizes of our included studies may limit the power to find positive association between SLE and GDM. Second, data about demographic characteristics (as body mass index and dietary characteristics) and clinical manifestations (as disease activity and drug usage), associated with GDM risk, was absent in our included studies, and differences of these characteristics across our original studies may account for the significant heterogeneity. Also, screening methods of GDM in our original studies were obscure, which may cause the results varying. Lastly, the significant heterogeneity may limit the generalizability of the pooled results.

## Conclusions

In summary, this meta-analysis suggested SLE is not associated with the risk of GDM and glucocorticoids therapy is associated with the increased risk of GDM in SLE patients. A right time for pregnancy (remission or inactive disease status) and detailed preconception counselling are pivotal for a successful pregnancy. Furthermore, further elaborate prospective cohort studies and immunologic researches are needed to reveal the mechanism underlying GDM and glucocorticoids use.

## Additional file

Additional file 1: **Table S1.** PRISMA checklist. **Table S2.** Characteristics about SLE patients of included studies. **Figure S1.** Search strategy of the electrical databases. **Figure S2.** Sensitivity analysis of meta-analysis (DOCX 512 kb)

## Abbreviations

ACR: American College of Rheumatology
anti-ds-DNA: Anti-double stranded DNA antibodies
aPL: Antiphospholipid antibody
APOs: Adverse pregnancy outcomes
APS: Antiphospholipid syndrome
CI: Confidence interval
GDM: Gestational diabetes mellitus
MOOSE: Meta-analysis of observational studies in epidemiology
NOS: Newcastle-Ottawa Scale
PRISMA: Preferred Reporting Items for Systematic Reviews and Meta-Analyses
RR: Risk ratios
SLE: Systemic lupus erythematosus
